# Development and validation of a nomogram for all-cause mortality in osteoporosis patients over five years

**DOI:** 10.1371/journal.pone.0334913

**Published:** 2025-10-16

**Authors:** Yuhao Li, Xiaowan Xie, Yazhou Liu, Zhaoqi Gong, Minglong Bi, Yuanyuan Li

**Affiliations:** 1 Department of Orthopedics, Dandong Central Hospital, China Medical University, Dandong, China; 2 Department of Oncology, Dandong Central Hospital, China Medical University, Dandong, China; 3 Department of Orthopedics, Dandong Central Hospital, Dalian Medical University, Dandong, China; 4 Department of General surgery, Dandong Central Hospital, China Medical University, Dandong, China; University of Padova: Universita degli Studi di Padova, ITALY

## Abstract

**Purpose:**

Osteoporosis significantly increases fracture risk and mortality, yet robust tools for predicting long-term mortality in this population are lacking.This study aimed to develop and validate a nomogram for predicting 5-year all-cause mortality among patients with osteoporosis.

**Methods:**

A retrospective cohort study was conducted using data from 2,165 osteoporosis patients sourced from the NHANES database (2007–2023; training set) and 304 patients from Dandong Central Hospital (2017–2024; validation set). Potential risk factors were analyzed through LASSO regression, followed by multivariate logistic regression, to identify independent predictors.A nomogram was constructed employing significant predictors. Finally, the C-index, ROC curve, calibration curve, and decision curve analysis were utilized to validate the model in both the training and validation sets.

**Results:**

In the study population, 192 patients died in the training set and 36 patients died in the experimental set. At the same time, we collected detailed baseline demographic data. Specifically, the age distribution of the training set was 56.07 ± 17.62, and that of the experimental set was 57.11 ± 18.34. Among them, 49.52% of the training set were male, and 50.99% of the experimental set were male. During the study period, we recorded 228 deaths. Seven independent predictors of 5-year all-cause mortality were identified: increased Age (OR=1.090,95%Cl: 1.115–2.313), Male gender (OR=1.606,95%Cl: 1.071–1.109), Smoking (OR=1.945,95%Cl: 1.289–2.933), higher FBG (OR=1.006,95%Cl: 1.002–1.010), higher Uric acid (OR=1.177,95%Cl: 1.039–1.332); Alcohol use (OR=0.583,95%Cl: 0.410–0.827) and higher BMI (OR=0.946,95%Cl: 0.909–0.985) were protective. The resulting nomogram demonstrated strong discriminatory ability in both the training set (AUC = 0.834) and validation set (AUC = 0.862). In the validation set, the precision rate was 0.514, the recall rate was 0.5, and the F1-score was 0.507. Calibration plots and the Hosmer-Lemeshow test indicated good agreement between predicted and observed outcomes (p > 0.05). Decision curve analysis confirmed significant clinical utility across a wide range of risk thresholds.

**Conclusion:**

This study developed and validated a novel nomogram incorporating seven common clinical factors, which can predict the 5-year all-cause mortality risk in patients with osteoporosis. Although the tool demonstrated good performance and has the potential to assist in clinical risk stratification and personalized management, there are still some limitations in the study design. Therefore, its clinical applicability should be interpreted with caution until further external validation.

## Introduction

Osteoporosis is widely acknowledged as the most prevalent metabolic bone disorder worldwide [1]. This condition is characterized by a reduction in bone density, deterioration of bone tissue, disruption of bone microarchitecture, compromised bone strength, and an increased risk of fractures [2]. It is estimated that approximately 200 million individuals are affected by osteoporosis globally, with the annual incidence of osteoporotic fractures surpassing 1.5 million in the United States alone [1,3,4]. At present, the likelihood of developing osteoporosis is greatest in North America and Europe; however, this prevalence is anticipated to rise in developing countries as life expectancy continues to improve in these regions [5].

It is essential to acknowledge that the most severe complication associated with osteoporosis is fracture. The social and economic burden imposed by osteoporotic fractures is progressively escalating due to the aging global population [1,6–8].Projections suggest that by 2050, the annual incidence of osteoporotic fractures in China is projected to approach approximately 5.99 million, with associated healthcare expenditures expected to escalate to $25.43 billion [9].

Despite its prevalence, a significant gap persists in understanding the factors contributing to all-cause mortality among individuals diagnosed with osteoporosis. Although numerous prognostic models for osteoporosis have been developed thus far, a considerable proportion of these models exhibit significant limitations concerning their predictive accuracy, generalizability, and clinical applicability [10–13]. These constraints frequently arise from issues such as inadequate sample sizes, insufficient consideration of multifactorial risk factors, and a lack of validation across heterogeneous populations.Consequently, there exists an urgent necessity for more robust and comprehensive predictive frameworks capable of effectively forecasting mid-to-long-term mortality in patients with osteoporosis.

This study aims to develop and validate a nomogram that predicts all-cause mortality in patients with osteoporosis over a five-year period. By integrating multiple clinical risk factors into a unified predictive model, we seek to enhance patient stratification and inform clinical decision-making. This nomogram is designed to provide clinicians with a valuable tool for assessing mortality risk, allowing healthcare providers to make more informed treatment decisions tailored to individual patient profiles. For instance, based on the risk stratification provided by the nomogram, clinicians may opt for more aggressive interventions in high-risk patients, such as initiating pharmacotherapy or increasing monitoring frequency.

The significance of this research lies not only in its potential to improve patient outcomes through targeted management but also in its contribution to a deeper understanding of osteoporosis-related mortality. This personalized assessment enhances clinical decision-making and empowers patients by providing them with a clearer understanding of their health risks, ultimately leading to improved engagement in their care plans. With further rigorous validation, this nomogram may have the potential to serve as a useful tool for clinicians, enabling more accurate risk prediction and enhancing the overall quality of care for patients with osteoporosis.

## Methods

### Data sources

This study utilized a retrospective cohort design based on data obtained from the National Health and Nutrition Examination Survey (NHANES), collected between 2007 and 2023. NHANES is a nationally representative, stratified multistage probability sample survey conducted by the National Center for Health Statistics (NCHS) [14], with further details accessible at http://www.cdc.gov/nchs/nhanes. The NCHS meticulously maintains and manages the database. Informed written consent was obtained from all participants, and the study received approval from the NCHS Institutional Review Board (IRB). Additional information regarding the IRB process can be found at http://www.cdc.gov/nchs/nhanes/irba98.htm. As NHANES is a publicly available database containing anonymized data, no further ethical approval or informed consent was required. This study strictly adhered to institutional guidelines and those established by data custodians to ensure the safety and privacy of human subjects.

Additionally, clinical data were collected from 304 patients diagnosed with osteoporosis at Dandong Central Hospital between October 2017 and October 2024. This study was conducted in accordance with the STROCSS (Strengthening the Reporting of Cohort Studies in Surgery) guidelines and strictly adhered to the ethical principles outlined in the Helsinki Declaration of 1964, along with its subsequent amendments. Approval was obtained from the Institutional Ethics Committee of our healthcare facility to ensure compliance with ethical standards. Written informed consent was obtained from all participants or their legal guardians prior to inclusion, thereby safeguarding the rights and welfare of the participants. Only clinical data were collected, ensuring the exclusion of personal identifiers. The study methodology was reviewed and approved ethically, and a waiver of informed consent was granted.

In this study, we utilized two datasets: the National Health and Nutrition Examination Survey (NHANES) and a hospital cohort of osteoporosis patients. The inclusion of both datasets enhances the robustness of our nomogram for predicting all-cause mortality in osteoporosis patients. NHANES provides a nationally representative sample with diverse ages, races, and socio-economic backgrounds, ensuring broad external validity. In contrast, the hospital cohort includes patients diagnosed with osteoporosis who are seeking medical care, often presenting more severe disease manifestations. By integrating these sources, we aim to assess the performance of our nomogram across different clinical scenarios, thus increasing its relevance for clinicians working with various patient demographics.

### Patient selection

The objective of this study was to develop and validate a predictive model for five-year all-cause mortality in patients diagnosed with osteoporosis. These participants were selected according to specific inclusion and exclusion criteria, which are outlined below.

**Fig 1:**
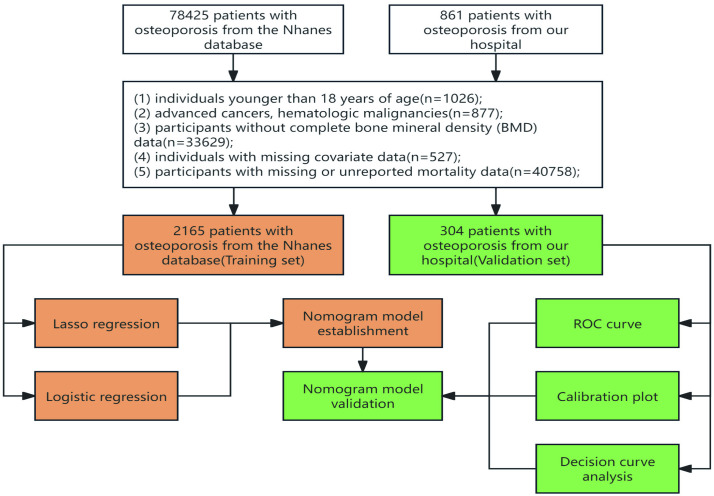
Flow diagram of study design.

The following inclusion criteria were applied: (1) a formal diagnosis of osteoporosis according to the criteria of the International Osteoporosis Foundation (IOF) or the World Health Organization (WHO); (2) a minimum of five years of follow-up to facilitate the assessment of all-cause mortality over this period. The exclusion criteria for this study were: (1) age younger than 18 years; (2) advanced cancers or hematologic malignancies; (3) incomplete bone mineral density (BMD) data; (4) missing covariate data; (5) missing or unreported mortality data.The screening process and participant flow are shown in a flow diagram in Fig 1.

### Measurement of OP

Osteoporosis diagnosis was established through bone mineral density (BMD) assessment via dual-energy X-ray absorptiometry (DXA), conducted by certified radiologic technologists using the Hologic QDR-4500 A fan-beam densitometer (Hologic, Bedford, Massachusetts, USA). All DXA data were processed and analyzed using Hologic APEX software (version 4.0). Detailed methodology can be found on the official NHANES website. Participants were categorized into three groups based on their BMD measurements at the total femur (TF), femoral neck (FN), or lumbar spine (LS): normal, osteopenia, or osteoporosis. The diagnostic criteria for osteoporosis followed the definitions established by Looker et al. [15,16]. Reference BMD values were derived from the mean BMD of young adults aged 18–25 years, representing the general male and female population. Osteoporosis was diagnosed in individuals with a BMD score 2.5 standard deviations below the reference value, while those with a BMD score within 1.0 standard deviation of the reference value were classified as having normal BMD.

### Definition of mortality

The primary outcome was the five-year risk of all-cause mortality in patients with osteoporosis. This risk was defined as death from any cause occurring within a five-year follow-up period following enrollment. By integrating NHANES data with the National Death Index (NDI) mortality records, accessible via public linkage as of December 31, 2019, we were able to determine mortality rates within the follow-up cohort. A probabilistic matching algorithm was employed to ensure accurate linkage. Furthermore, the classification of causes of death adhered to the International Classification of Diseases, Tenth Revision (ICD-10) [17]. Information on deaths among clinical patients was obtained from official medical records, death certificates, or national mortality databases, ensuring data accuracy. Censoring was applied to patients who were lost to follow-up.

### Variables

Potential risk factors associated with osteoporosis were identified through a comprehensive literature review and a thorough examination of patient records. The data collected included variables such as age, gender, self-reported diabetes, self-reported hypertension, smoking status, alcohol consumption, and physical activity levels, all of which were carefully selected to minimize potential confounding bias. Demographic information, including age and gender, was gathered by trained interviewers using a computer-assisted personal interview (CAPI) system, which facilitated the administration of household and population-based surveys.

Participants were categorized into smoking status groups based on their responses to survey questions (SMQ020: “Have you ever smoked 100 cigarettes?” and SMQ040: “Do you currently smoke?”). Individuals were classified as never smokers if they had never smoked 100 cigarettes in their lifetime and were not currently smoking. Current smokers were defined as those who had smoked 100 or more cigarettes and continued to smoke at the time of the survey. Former smokers were individuals who had smoked 100 or more cigarettes but had since ceased smoking. For the purposes of this study, both never smokers and former smokers were categorized as current nonsmokers.

Alcohol consumption was similarly categorized into four groups: heavy drinkers, moderate drinkers, light drinkers, and non-drinkers, based on self-reported frequency of alcohol intake. Heavy drinkers were defined as individuals who reported consuming four or more drinks per day for men (or five or more for women), moderate drinkers consumed three or fewer drinks per day, and light drinkers had a history of alcohol consumption but reported fewer than 12 drinks in the past year. For the purposes of this study,non-drinkers were defined as individuals who reported never having consumed alcohol, while all others were classified as drinkers.

Diabetes and hypertension status were assessed using a combination of self-reported questionnaire responses and laboratory data to enhance diagnostic accuracy. Diabetes status was determined based on responses to the following survey questions: “Has a healthcare provider ever told you that you have diabetes?”, “Do you use insulin?”, and “Do you use oral hypoglycemic agents?”. Laboratory criteria for diabetes included a fasting blood glucose level ≥ 7.0 mmol/L, HbA1c level ≥ 6.5%, and a glucose level ≥ 11.1 mmol/L following an oral glucose tolerance test (OGTT). Similarly, hypertension was diagnosed based on either multiple blood pressure readings ≥ 130/80 mmHg or a self-reported physician diagnosis of hypertension.

To ensure the consistency of predictor variables between the two datasets, we first ensured that the definitions of these variables were aligned. Clinical measurements such as bone mineral density, age, and gender were defined using standardized criteria in accordance with relevant clinical guidelines. Prior to analysis, we performed rigorous data preprocessing to identify and resolve any inconsistencies in variable coding and measurement units. This included converting categorical variables into uniform categories and standardizing continuous variables for consistency. Additionally, we conducted pilot testing and validation. To further verify the standardization process, we performed pilot tests on key variables from both datasets. Any inconsistencies identified during this phase were addressed through additional adjustments, ensuring that the final dataset used for analysis exhibited a high degree of compatibility between the two sources.

### Statistical analysis

Descriptive statistics were employed to summarize the data. Categorical variables were presented as frequencies and percentages (%) and compared using Chi-square tests. Continuous variables were expressed as means ± standard deviations and analyzed using independent sample t-tests.

In the training cohort, Least Absolute Shrinkage and Selection Operator (LASSO) regression was utilized for variable selection, followed by multivariate logistic regression to identify independent predictors of all-cause mortality in patients with osteoporosis.

A nomogram to predict the risk of all-cause mortality was developed using R software (The R Foundation for Statistical Computing, Vienna, Austria), incorporating variables identified through the multivariate logistic regression model derived from the training dataset.

The performance of the predictive nomogram was evaluated by constructing a receiver operating characteristic (ROC) curve and calculating the area under the curve (AUC) to assess the model’s discriminative ability in terms of sensitivity and specificity. The optimal cutoff value was determined using the Youden index. Variance inflation factors (VIFs) were calculated to evaluate multicollinearity within the multivariate logistic regression model. Model calibration was assessed using the Hosmer–Lemeshow goodness-of-fit test to evaluate the accuracy of the nomogram. Decision curve analysis (DCA) was performed to determine the clinical utility of the model by quantifying the net benefit across a range of threshold probabilities.

To comprehensively evaluate the model’s performance, ROC analysis, calibration plots, and DCA were conducted on both the training and validation datasets. All statistical analyses were performed using SPSS version 26.0 (IBM Corp., Armonk, NY, USA) and R version 4.0.3 (The R Foundation for Statistical Computing, Vienna, Austria).

## Results

### Baseline clinical and demographic characteristics of patients

In this study, we identified a total of 2,165 patients diagnosed with osteoporosis from our database, forming the training set, along with 304 patients clinically diagnosed with osteoporosis, constituting the validation set.“Clinical diagnosis” is based on a physician’s evaluation and relevant symptoms, while “confirmed diagnosis” refers to standardized diagnostic criteria established through imaging techniques such as dual-energy X-ray absorptiometry (DXA).Among them, 192 patients died in the training set, and 36 patients died in the experimental set. The training set was utilized to develop our predictive model. Notably, as shown in Table 1, significant differences were observed between the two groups regarding various baseline clinical and demographic characteristics, including the prevalence of diabetes mellitus and levels of triglycerides, high-density lipoprotein (HDL), blood urea nitrogen (BUN), creatinine (Cr), total bilirubin (STB), uric acid, and calcium (p < 0.05). We also compared the clinical and demographic characteristics of patients who survived versus those who died in both the training and validation sets(as shown in Table 2).

**Table 1:** Baseline clinical and demographic characteristics of the training and validation set.

Variables	Training set (n = 2165)	Validation set (n = 304)	p-value
Demographic			
Age, years	56.07 ± 17.62	57.11 ± 18.34	0.337
Male gender (n,%)	1072 (49.52%)	155 (50.99%)	0.631
Smoking (n,%)	706 (32.61%)	100 (32.89%)	0.921
Alcohol use (n,%)	1689 (78.01%)	239 (78.62%)	0.811
Comorbidities			
Hypertension (n,%)	824 (38.06%)	109 (35.86%)	0.458
Diabetes (n,%)	291 (13.44%)	65 (21.38%)	<0.001
Laboratory findings			
BMI kg/m^2^	26.61 ± 5.03	26.27 ± 4.68	0.493
FBG (mg/dL)	109.85 ± 37.71	111.62 ± 40.31	0.059
Triglyceride (mg/dL)	135.20 ± 119.11	137.14 ± 75.53	0.004
HDL (mg/dL)	55.54 ± 16.37	46.17 ± 11.70	<0.001
LDL (mmol/l)	3.02 ± 0.94	3.01 ± 0.94	0.814
BUN (mg/dL)	14.07 ± 6.44	17.88 ± 6.10	<0.001
Cr (μmol/L)	0.91 ± 0.55	0.71 ± 0.23	<0.001
P (mg/dL)	3.69 ± 0.55	3.63 ± 0.69	0.139
STB (mg/dL)	0.82 ± 0.29	0.92 ± 0.63	0.036
Uric acid (μmol/L)	5.47 ± 1.41	5.64 ± 1.42	0.021
Calcium (mmol/l)	2.35 ± 0.09	2.22 ± 0.15	<0.001

SD, standard deviation; BMI, body mass index; FBG, fasting blood glucose; HDL, high-density lipoprotein; LDL, low-density lipoprotein; BUN, blood urea nitrogen; Cr, creatinine ratio; P, phosphorus; STB, serum total bilirubin.

p < 0.05: statistically significant difference.

**Table 2:** Clinical and demographic characteristics of patients who survived and died of all-cause death within 5 years in the training and validation sets.

Variables	Training set (n = 2165)			Validation set (n = 304)		
	Survive (n = 1973)	Death (n = 192)	p-value	Survive (n = 268)	Death (n = 36)	p-value
Demographic						
Age,years	54.51 ± 17.41	72.05 ± 10.43	**<0.001**	54.80 ± 18.06	74.28 ± 8.89	**<0.001**
Male gender (n,%)	960 (48.66%)	112 (58.33%)	0.010	141 (52.61%)	14 (38.89%)	0.122
Smoking (n,%)	658 (33.35%)	48 (25.00%)	0.018	93 (34.70%)	7 (19.44%)	0.067
Alcohol use (n,%)	1572 (79.68%)	117 (60.94%)	**<0.001**	219 (81.72%)	20 (55.56%)	**<0.001**
Comorbidities						
Hypertension (n,%)	704 (35.68%)	120 (62.50%)	**<0.001**	98 (36.57%)	11 (30.56%)	0.480
Diabetes (n,%)	239 (12.11%)	52 (27.08%)	**<0.001**	60 (22.39%)	5 (13.89%)	0.243
Laboratory findings						
BMI kg/m^2^	26.64 ± 5.02	26.31 ± 5.11	0.375	26.44 ± 4.58	24.98 ± 5.27	0.114
FBG (mg/dL)	108.49 ± 36.69	123.82 ± 44.66	**<0.001**	108.54 ± 32.62	134.58 ± 73.05	**<0.001**
Triglyceride (mg/dL)	135.14 ± 122.10	135.89 ± 82.51	0.933	136.38 ± 77.09	142.80 ± 63.35	0.632
HDL (mg/dL)	55.62 ± 16.29	54.69 ± 17.14	0.455	46.44 ± 11.78	44.20 ± 11.01	0.283
LDL (mmol/l)	3.04 ± 0.93	2.89 ± 0.97	0.030	3.02 ± 0.94	2.90 ± 0.95	0.492
BUN (mg/dL)	13.65 ± 5.61	18.44 ± 11.15	**<0.001**	17.99 ± 6.06	17.07 ± 6.45	0.400
Cr (μmol/L)	0.88 ± 0.47	1.21 ± 1.02	**<0.001**	0.71 ± 0.22	0.73 ± 0.27	0.702
P (mg/dL)	3.68 ± 0.54	3.73 ± 0.66	0.226	3.62 ± 0.69	3.66 ± 0.77	0.771
STB (mg/dL)	0.82 ± 0.29	0.83 ± 0.30	0.557	0.92 ± 0.65	0.96 ± 0.44	0.714
Uric acid (μmol/L)	5.41 ± 1.37	6.01 ± 1.72	**<0.001**	5.60 ± 1.33	5.91 ± 1.93	0.229
Calcium (mmol/l)	2.35 ± 0.09	2.35 ± 0.10	0.283	3.04 ± 13.43	2.22 ± 0.16	0.657

SD, standard deviation; BMI,body mass index; FBG, fasting blood glucose; HDL, high-density lipoprotein; LDL, low-density lipoprotein; BUN, blood urea nitrogen; Cr, creatinine ratio; P, phosphorus; STB, serum total bilirubin.

p < 0.05: statistically significant difference.

### Independent risk factors in the training set

**Fig 2:**
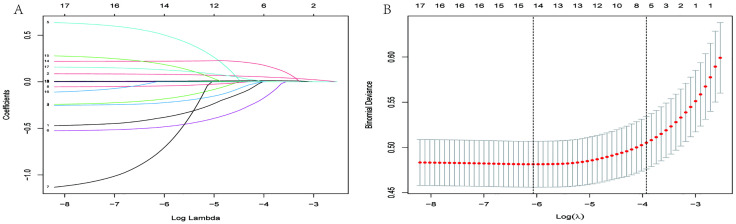
LASSO coefficient profiles of the 17 features. (A)A coefficient profile plot was produced against the log (lambda) sequence. (B) The partial likelihood deviance (binomial deviance) curve was plotted vs. log (lambda). Dotted vertical lines were drawn at the optimal values by using the minimum criteria and the 1 SE of the minimum criteria (the 1-SE criteria). LASSO performs shrinkage and selection on coefficients using the regularization parameter lambda.


In the training set, a total of 17 predictor variables were initially included, after which the LASSO regularization method was applied to identify 14 potential risk factors (as illustrated in Figs 2A and 2B). These factors were subsequently assessed through multivariable logistic regression analysis (Fig 2B). Following multivariable adjustment, 7 independent risk factors associated with all-cause mortality over five years in osteoporosis patients were identified (for the detailed selection process, see S1-Fig): age (OR = 1.090, 95% CI: 1.115–2.313, p = 0.011), male gender (OR = 1.606, 95% CI: 1.071–1.109, p < 0.001), smoking (OR = 1.945, 95% CI: 1.289–2.933, p = 0.002), alcohol use (OR = 0.583, 95% CI: 0.410–0.827, p = 0.003), BMI (OR = 0.946, 95% CI: 0.909–0.985, p = 0.006), FBG (OR = 1.006, 95% CI: 1.002–1.010, p = 0.003), and uric acid (OR = 1.177, 95% CI: 1.039–1.332, p = 0.010) (Table 3). Table 3 presents the results of the multivariate logistic regression analysis, including the intercept, standard error, β coefficients, and odds ratios. A new predictive formula was developed based on these regression coefficients and constants:

**Table 3:** Multivariate analysis of all-cause mortality over 5 years in patients with osteoporosis in the training set.

Risk factor	β	S.E	Wald	OR	95%Cl	P-valve
Age, years	0.086	0.009	96.279	1.090	1.115-2.313	**0.011**
Male gender	0.474	0.186	6.478	1.606	1.071-1.109	**<0.001**
Smoking	0.665	0.210	10.063	1.945	1.289-2.933	**0.002**
Alcohol use	−0.540	0.179	9.128	0.583	0.410-0.827	**0.003**
Hypertension	0.253	0.183	1.912	1.288	0.900-1.846	0.167
Diabetes	0.272	0.233	1.361	1.313	0.831-2.073	0.243
Calcium	−1.229	0.937	1.718	0.293	0.047-1.837	0.190
BMI	−0.055	0.020	7.449	0.946	0.909-0.985	**0.006**
FBG	0.006	0.002	8.590	1.006	1.002-1.010	**0.003**
Triglyceride	−0.001	0.001	0.531	0.999	0.997-1.001	0.466
BUN	0.004	0..013	0.082	1.004	0.978-1.030	0.775
Cr	0.222	0.130	2.900	1.248	0.967-1.611	0.089
P	0.284	0.161	3.102	1.329	0.968-1.823	0.078
Uric acid	0.163	0.063	7.550	1.177	1.039-1.332	**0.010**
Constant	−6.667	2.426	7.550	0.001	NA	**0.006**

β, beta; SE, standard error; Wald: Wald statistic; OR, odds ratio; CI, confidence interval; P, phosphorus;

BMI, body mass index; FBG, fasting blood glucose; BUN, blood urea nitrogen; Cr, creatinine ratio.

The p-value was used to determine whether the relationship between each independent variable and all-cause mortality in osteoporosis patients over a 5-year period was statistically significant.

Logit(p) = -6.667 + 0.086*Age+ 0.474*Male gender+ 0.665*Smoking-0.540*Alcohol use -0.055*BMI +0.006 *FBG + 0.163*uric acid.

$$\begin{array}{cc} \text{Logit}(\text{p})\;= & -\text{6}.\text{667}\;+\;\text{0}.086*\text{Age}\;+\;\text{0}.\text{474}*\text{Male}\;\text{gender}+\;\text{0}.\text{665}*\text{Smoking}-\text{0}.\text{540}*\text{Alcohol}\;\text{use}\;\\ & -\text{0}.\text{055}*\text{BMI}\;+\;\text{0}.\text{006}\;*\text{FBG}\;+\;\text{0}.\text{163}*\text{uric}\;\text{acid} \end{array}$$

### Nomogram model establishment

**Fig 3:**
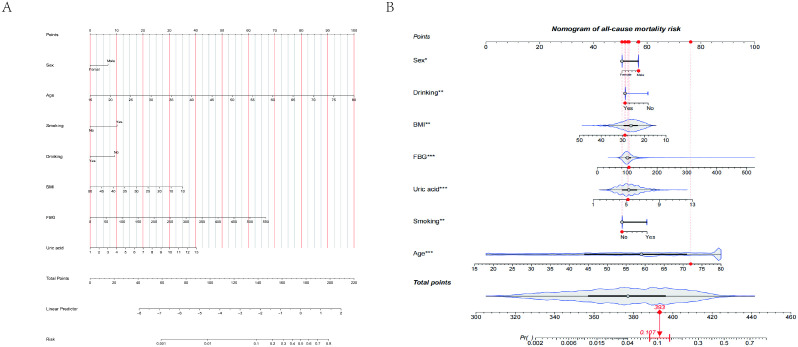
Nomogram for predicting all-cause mortality within 5 years in old osteoporotic patients. (A)Seven variables were included in the nomogram prediction model, namely: sex, age, smoking, drinking, BMI, FBG and uric acid. (B) Dynamic nomogram as an example. Caption credit: The significance of the asterisks beside each variable in part B represents the importance of all the risk factors. A simple example analysis:For example, a 55-year-old male patient (52 points), (57 points), without using alcohol (60 points), with smoking (60 points), with BMI = 30 kg/m2 (51 points), with uric acid = 9 μmol/L (65 points), and with FBG = 300 mg/dL (75 points) would have a total score of 420 points. This corresponds to a predicted five-year risk of all-cause mortality in osteoporosis patients of 40.33%.

A nomogram model was developed using multivariate logistic regression to estimate the individualized five-year risk of all-cause mortality in patients with osteoporosis. As illustrated in Fig 3A and 3B, this model incorporated seven independent risk factors. Each factor was assigned a specific score, and the cumulative score corresponded to the predicted five-year risk of all-cause mortality for individuals with osteoporosis. For example, a 55-year-old male patient (52 points) (57 points), without using alcohol (60 points), with smoking (60 points), with BMI = 30 kg/m2 (51 points), with uric acid = 9 μmol/L (65 points), and with FBG = 300 mg/dL (75 points) would have a total score of 420 points. This corresponds to a predicted five-year risk of all-cause mortality in osteoporosis patients of 40.33%.

### Nomogram model validation

**Fig 4:**
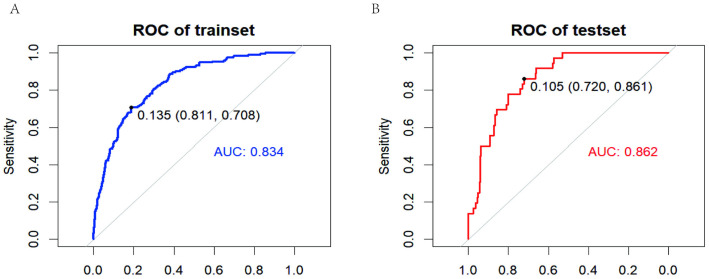
Receiver operating characteristic curves (ROC) of the training set (A) and validation set (B).

The nomogram achieved high area under the curve (AUC) values of 0.834 and 0.862 in the training and validation sets, respectively (Figs 4A and 4B). With a C-index of 0.834 (95% CI: 0.708–0.811), the nomogram demonstrated excellent discriminatory ability. At the same time, we measured an accuracy rate of 0.514, a recall rate of 0.5, and an F1 score of 0.507 on the validation set.

**Fig 5:**
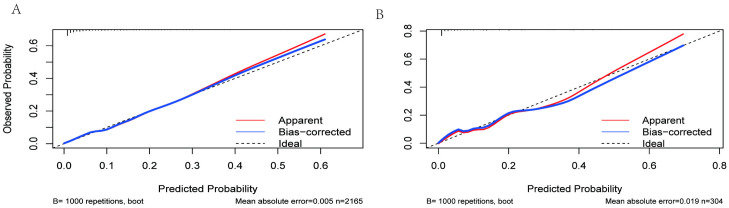
Calibration curve of constructed nomogram in the training set (A) and validation set (B). Caption credit: Predictions generated from the model are plotted against actual patient outcomes. The dotted line represents the perfect model calibration. The red line (apparent) indicates calibration when the model is applied to each set, and the blue line (bias-corrected) indicates calibration when the model is applied to the bootstrap set.

The variance inflation factors (VIFs) for the seven risk factors ranged from 1.051 to 1.161, indicating the absence of multicollinearity. Calibration plots revealed strong agreement between the predicted and observed probabilities (Figs 5A and 5B). The Hosmer-Lemeshow goodness-of-fit test showed no evidence of poor fit for the multivariate logistic regression models in both the training set (χ² = 6.449, df = 8, p = 0.597) and the validation set (χ² = 4.417, df = 8, p = 0.818), indicating that the models were well-calibrated.

**Fig 6:**
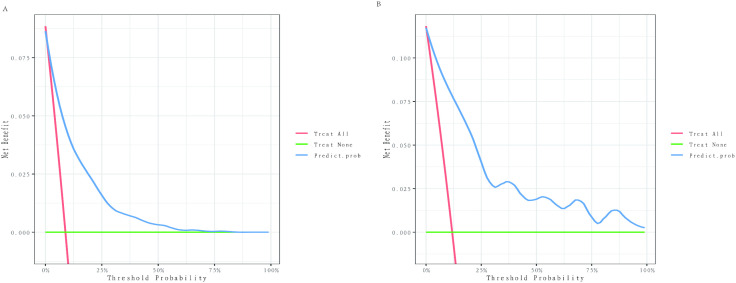
Decision curve analysis of the nomogram in the training set (A) and validation set (B). Caption credit: The blue line shows the net benefit of our model. The red line assumes that all patients will experience all-cause mortality within 5 years. The green line assumes that no patients experience an all-cause death within 5 years.

Decision curve analysis demonstrated that the nomogram consistently provided a superior net benefit compared to no assessment across a wide range of threshold probabilities (1–73% in the training set, 1–100% in the validation set) (Figs 6A and 6B).

## Discussion

Osteoporosis affects a significant proportion of individuals across all racial groups and is projected to emerge as a major global health challenge as the world’s population ages at an unprecedented rate [18]. The nomogram exhibited strong discriminatory power and predictive accuracy for estimating the five-year risk of all-cause mortality in patients with osteoporosis, as indicated by the validation metrics. These findings suggest that the proposed model can effectively support clinicians in making individualized treatment decisions based on a patient’s risk profile.The significance of this research lies not only in its potential to improve patient outcomes through targeted management but also in its contribution to a deeper understanding of osteoporosis-related mortality. Through rigorous validation, this nomogram is expected to serve as a crucial tool for clinicians, enabling more accurate risk prediction and enhancing the overall quality of care for patients with osteoporosis.The early optimization of osteoporosis assessment tools for enhanced prevention holds substantial academic and clinical value [19,20]. Nomograms provide more accurate and comprehensible prognostic assessments compared to conventional staging methods and are now widely utilized as prognostic tools in medicine [21–23].

To explore the predictors of five-year all-cause mortality risk in osteoporosis patients, this study developed a nomogram containing several clinical and laboratory parameters by using database data as a training set and real-world clinical data as a validation set. These parameters are strongly associated with the long-term survival outcomes of osteoporosis patients. Through statistical analysis and multivariate logistic regression, we assigned weighted coefficients to seven variables: Age, Male gender, Smoking, Alcohol use, BMI, FBG, and uric acid. However, it is important to acknowledge that the reliance on retrospective data may introduce biases that could affect the generalizability of our findings. Additionally, the low prevalence of mortality events in our cohort raises concerns about the robustness of our predictive model; thus, further validation in diverse populations is warranted. Despite these limitations, his is the first nomogram specifically developed to predict the five-year all-cause mortality risk in patients with osteoporosis. The nomogram utilizes a set of variables and corresponding coefficients to offer an intuitive risk assessment tool. It equips clinicians with a reliable means of identifying risk factors associated with mortality, enabling more effective identification of high-risk patients and the formulation of personalized treatment and intervention strategies.

### Age

The findings of this study indicate that age serves as a significant predictor of all-cause mortality risk in patients with osteoporosis. This conclusion aligns with existing literature, which demonstrates that numerous studies have identified a substantial increase in all-cause mortality among osteoporosis patients as age advances [24,25].

Advanced age is recognized as an independent risk factor for five-year all-cause mortality in elderly patients with osteoporosis. This association may be attributed to various pathological and physiological changes associated with aging. Firstly, elderly patients are often accompanied by multiple chronic diseases, such as cardiovascular disease, diabetes, and neurodegenerative conditions; these comorbid states may exacerbate the risk of mortality. Secondly, with advancing age, physiological functions gradually decline, particularly impacting the health of the skeletal system [26]. In the elderly population, there is a progressive reduction in bone density and an increased incidence of bone fractures, further compounded by a diminished capacity for rehabilitation following a fracture, thereby elevating the risk of mortality. Additionally, muscle atrophy (i.e., sarcopenia), which is prevalent among the elderly, adversely affects exercise capacity and overall health, consequently increasing the risk of death [27,28]. Finally, addressing these factors through comprehensive geriatric assessments and multidisciplinary interventions may contribute to reducing the five-year risk of all-cause mortality in this high-risk population.

### Male gender

The results of this study demonstrated that the all-cause mortality rate among male patients with osteoporosis was significantly higher than that in female patients. This finding is consistent with numerous epidemiological studies, which suggest that men have a distinct role in the morbidity and mortality associated with osteoporosis [24,29–31].

Although women face an increased risk of osteoporosis due to the decline in estrogen levels following menopause [32], men tend to exhibit higher mortality rates from osteoporosis-related complications [33,34].This may be attributed to significant differences in bone density changes, bone metabolism, and hormone levels between the sexes. Men typically experience a more pronounced loss of bone density at an older age but tend to have poorer recovery and prognosis following fractures [31,34]. Moreover, although men generally possess higher muscle mass, studies have shown that older men often face an elevated risk of muscle atrophy, which may further exacerbate their risk of mortality in the context of osteoporosis [35]. Additionally, men may engage in higher-risk behaviors, such as smoking and alcohol consumption, which, combined with osteoporosis, increase their mortality risk [36,37].Therefore, when developing interventions, clinicians should emphasize the importance of screening, early intervention, and long-term management for male patients.

### Smoking

The results of this study demonstrated that smoking significantly increases the risk of all-cause mortality in patients with osteoporosis. This finding aligns with numerous epidemiological studies suggesting that smoking is not only associated with the development of osteoporosis but also adversely affects the survival of individuals already diagnosed with the disease [36,38,39]. Smoking may be detrimental to bone health through several mechanisms, thereby heightening the risk of mortality.

Firstly, nicotine and other harmful substances may impair osteoblast function, leading to decreased bone density and increased bone resorption [40,41]. Secondly, smoking may result in reduced estrogen levels, which could have a more pronounced impact on women, particularly postmenopausal patients [42,43]. Finally, smoking has been linked to alterations in the synthesis and metabolism of vitamin D, potentially decreasing the efficiency of calcium absorption and further exacerbating osteoporosis [44].

Additionally, smokers exhibit a significantly elevated risk of fractures among patients with osteoporosis, which is a major contributor to mortality. Research indicates that smokers experience higher rates of postoperative complications and increased mortality following hip fractures [45]. This phenomenon may be attributed to the adverse effects of smoking on wound healing, lung function, and overall health; thus, the potential consequences of fractures should not be underestimated when assessing the impact of smoking on mortality risk in osteoporosis patients. Collectively, these factors may further amplify the risk of death in this population. Therefore, conducting a comprehensive health assessment of patients who smoke is crucial when devising interventions aimed at reducing mortality risk.

### Alcohol use

The present study demonstrated that a history of alcohol consumption was inversely associated with the risk of mortality in patients with osteoporosis, a finding that aligns with certain phenomena described in the literature [46,47]. While it is widely accepted that excessive alcohol consumption negatively impacts bone health, moderate alcohol intake may exert a protective effect, offering new insights into the survival of patients with osteoporosis. This may be due to the fact that moderate alcohol consumption can promote bone health by enhancing circulation and reducing bone resorption. Additionally, moderate alcohol consumption may help alleviate stress, improve the overall quality of life in older adults, and reduce the incidence of psychological issues, such as depression and anxiety, which could potentially influence their survival rates.

Although the present study found that a history of alcohol consumption was inversely associated with the risk of mortality in osteoporosis patients, it is important to emphasize that this does not imply that uncontrolled alcohol consumption should be encouraged. The potential benefits of moderate alcohol consumption should be considered within the framework of personalized medicine, particularly when managing elderly osteoporotic patients. Future studies should further investigate the specific effects of various types and quantities of alcohol consumption on the survival of osteoporosis patients, as well as the underlying mechanisms, in order to develop evidence-based intervention strategies.

### BMI

This study investigated the relationship between body mass index (BMI) and the risk of mortality in patients with osteoporosis, revealing a significant association. Specifically, lower BMI may be correlated with an increased risk of mortality among osteoporosis patients, a finding that is consistent with several observations in the existing literature [48]. Low BMI is frequently regarded as a risk factor for osteoporosis, as underweight individuals often experience reduced bone mineral density and decreased bone mass, both of which contribute to a higher risk of fractures and related complications. In older adults, low BMI not only signifies diminished bone mass but may also be associated with malnutrition and muscle atrophy, further exacerbating health risks and impacting survival. Conversely, while a higher BMI may confer some degree of bone protection, excessive weight can similarly precipitate a variety of health issues, including cardiovascular disease and diabetes. These chronic conditions may adversely affect the overall health status of elderly patients, thereby influencing their survival rates. Consequently, it is essential to identify a balance between high and low BMI to establish an optimal weight range. Given the impact of BMI on mortality risk in osteoporosis patients, clinical attention should focus on the assessment and management of patients’ weight. Regular monitoring of BMI, combined with tailored nutritional guidance and exercise programs, can effectively enhance bone health and improve overall quality of life.

### FBG

This study investigated the relationship between fasting blood glucose (FBG) levels and the risk of mortality in patients with osteoporosis. The results indicate that elevated fasting blood glucose levels may be significantly associated with an increased risk of mortality among osteoporosis patients, providing new insights into the effects of abnormal glucose metabolism on bone health and overall survival.

Elevated fasting blood glucose (FBG) may influence the onset and progression of osteoporosis through various mechanisms. Research has demonstrated that heightened fasting blood glucose can impair the function of osteoblasts and osteoclasts, promote bone resorption, and inhibit bone formation, thereby exacerbating osteoporosis [49]. Furthermore, hyperglycemia individuals are at a higher risk of fractures [50]. The increased risk may be due to microvascular complications associated with hyperglycemia, peripheral nerve damage, and disturbances in bone metabolism, all of which contribute to decreased bone density and impaired bone quality. [51]. Consequently, in osteoporosis patients, elevated fasting blood glucose levels not only indicate potential issues with glucose metabolism but may also reflect greater systemic health risks. Given the impact of fasting blood glucose levels on mortality risk in osteoporosis patients, regular monitoring of FBG should be regarded as a crucial component of clinical management. Comprehensive strategies, including effective glycemic control, lifestyle modifications, and improved nutritional intake, are recommended in treatment regimens to mitigate the incidence of osteoporosis and enhance patient survival outcomes.

### Uric acid

The results of this study suggest that elevated uric acid levels may be significantly associated with increased mortality in patients with osteoporosis. Uric acid is the final metabolic product of purine metabolism and serves an antioxidant role in the body [52]. In patients with osteoporosis, elevated uric acid levels may influence bone metabolism and exacerbate bone loss by inducing oxidative stress and pro-inflammatory responses [53]. Furthermore, high uric acid levels may enhance osteoclast activity and inhibit osteoblast function, thereby reducing bone quality and further increasing fracture risk and mortality [54]. Given the potential impact of uric acid levels on mortality risk in osteoporosis patients, regular monitoring of uric acid levels should be incorporated into clinical assessments. Additionally, interventions, including dietary modifications, lifestyle changes, and pharmacological treatments, should be considered as part of a comprehensive strategy to control uric acid levels, improve bone health, and reduce the risk of mortality.

Our study offers valuable insights into the roles of age, smoking, and alcohol consumption in osteoporosis management, highlighting their potential as predictive factors for risk stratification. This understanding enables healthcare professionals to implement targeted interventions, such as lifestyle modification programs focusing on smoking cessation and responsible alcohol use, thus improving patient outcomes. Additionally, our findings stress the importance of educating patients about how their lifestyle choices impact bone health, empowering them to make informed decisions. Furthermore, we advocate for continued research to explore the mechanisms behind these associations, which may uncover new therapeutic targets to enhance patient care.

## Limitations

Nomograms are emerging as valuable noninvasive visualization tools for clinical prediction models. In this study, we developed and validated a nomogram to predict the five-year risk of all-cause mortality in patients with osteoporosis, utilizing database data as the training set and real clinical data as the validation set. The model has the potential to serve as an effective clinical decision-making tool.

However, several limitations must be considered when interpreting the results. First, this study employed a retrospective cohort design, which, while leveraging a substantial amount of real clinical and database data, is inherently susceptible to selection and information biases. These biases can arise from inconsistent data collection methods, incomplete patient records, or variations in diagnostic criteria across different institutions. Such factors may lead to an overestimation or underestimation of the associations identified in our analysis.Second, the training and validation sets were derived from different sources, resulting in differences in baseline characteristics, disease severity, and observation periods between the two patient groups. This discrepancy may affect the external validity of the model, as it raises concerns about whether the findings are applicable to other populations or clinical settings. Variations in patient demographics or treatment protocols between sites could influence mortality outcomes, thereby limiting the model’s generalizability.Third, several potential confounders were not included in the model, such as lifestyle factors (e.g., physical activity levels, dietary habits), psychosocial variables (e.g., mental health status, social support), and detailed comorbid conditions that could impact mortality risk. The exclusion of subjects with fragility fractures and BMD values consistent with osteopenia from the diagnosis of osteoporosis may limit the comprehensiveness of our findings.Finally, the relatively small size of the validation set undermines the robustness of the model. A limited sample size reduces the statistical power of our findings and may lead to overfitting, where the model performs well on the training data but fails to generalize to other datasets. Future studies should aim to conduct further validation using larger, multicenter datasets that encompass diverse populations. This approach would enhance the generalizability and reliability of the nomogram, ultimately providing a more accurate tool for predicting all-cause mortality in osteoporosis patients.By acknowledging these limitations, we hope to provide a more comprehensive understanding of the context in which our findings should be interpreted and to inform future research directions in this important area of study.

## Conclusion

In this study, we developed and validated a novel nomogram for predicting the five-year risk of all-cause mortality in patients with osteoporosis. The nomogram demonstrates strong discrimination, calibration, and clinical utility, and may aid in identifying high-risk patients for timely interventions aimed at reducing the risk of preventable mortality. However, further external validation is required prior to its clinical application.

## Supporting information

S1 Fig Variable Selection Process for Osteoporosis Mortality Risk Factors. (DOCX)

## Abbreviations

CI: Confidence Interval
OR: Odds Ratio
OP: Osteoporosis
NHANES: National Health and Nutrition Examination Survey
NCHS: National Center for Health Statistics
DXA: Dual energy X-ray absorptiometry
VIFs: Variance inflation factors
DCA: Decision curve analysis
ROC: receiver operating characteristic
AUC: area under curve
LASSO: Least Absolute Shrinkage and Selection Operator
FBG: fasting blood glucose
Cr: creatinine.
