# Supplementary figures and images for: Development and validation of a nomogram for all-cause mortality in osteoporosis patients over five years
Source: PLoS One. 2025 Oct 16;20(10):e0334913. doi: 10.1371/journal.pone.0334913 (PMC12530536; doi:10.1371/journal.pone.0334913)

**S1 Fig.** Variable Selection Process for Osteoporosis Mortality Risk Factors


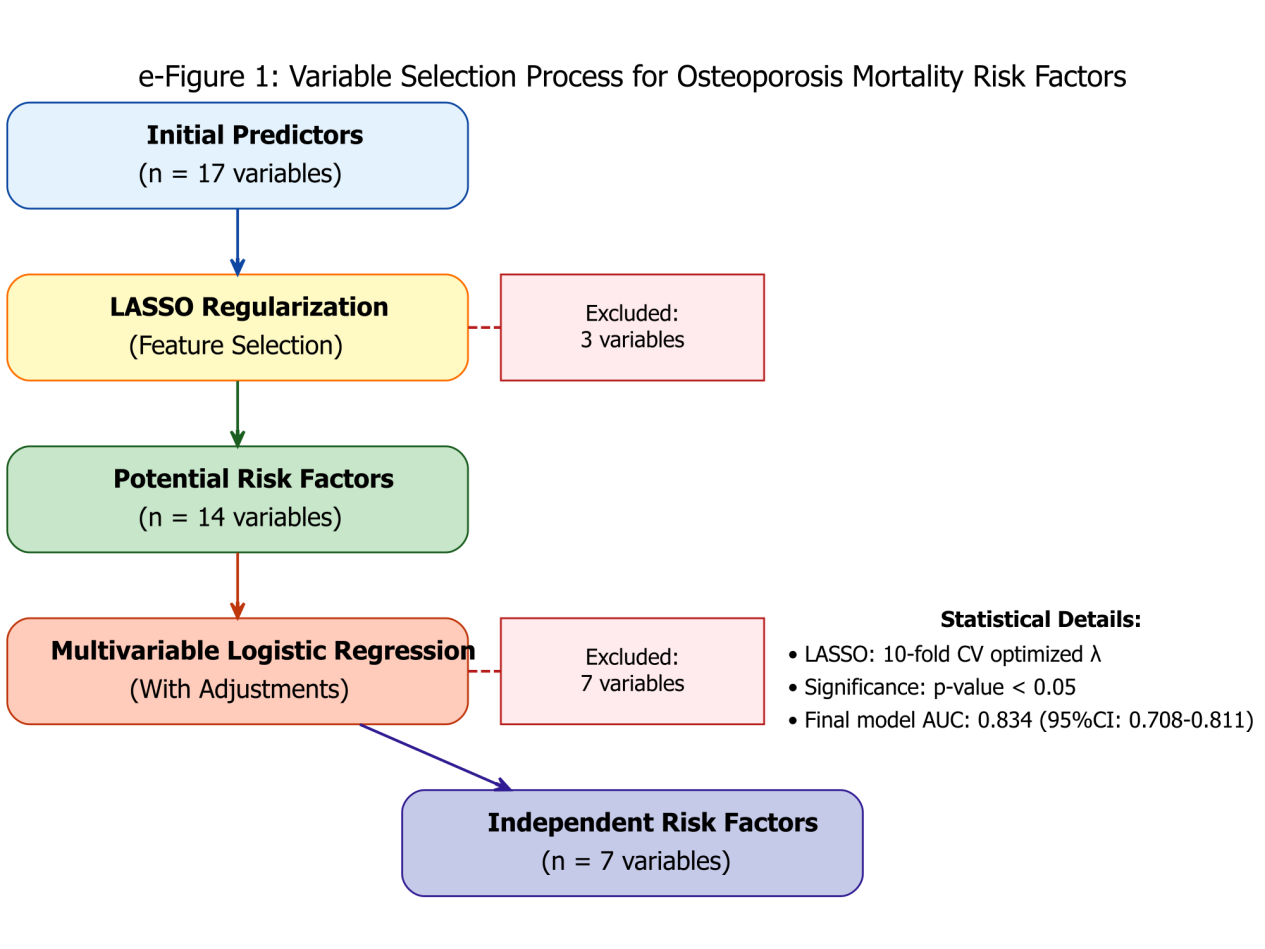

Supplement: S1 Fig — (DOCX) [file pone.0334913.s001.docx]
